# Risk factors for oropharyngeal dysphagia in cardiovascular diseases

**DOI:** 10.1590/1678-7757-2019-0489

**Published:** 2020-05-11

**Authors:** Tatiana Magalhães de ALMEIDA, Lívia Maria Silva GOMES, Débora AFONSO, Daniel MAGNONI, Isabela Cardoso Pimentel MOTA, João Ítalo Dias FRANÇA, Roberta Gonçalves da SILVA

**Affiliations:** 1 Instituto Dante Pazzanese Instituto de Cardiologia Departamento de Fonoaudiologia São PauloSP Brasil Instituto Dante Pazzanese , Instituto de Cardiologia , Departamento de Fonoaudiologia , São Paulo , SP , Brasil .; 2 Universidade Estadual Paulista Faculdade de Filosofia e Ciências Departamento de Fonoaudiologia MariliaSP Brasil UNESP - Universidade Estadual Paulista , Faculdade de Filosofia e Ciências , Departamento de Fonoaudiologia , Marilia , SP , Brasil .; 3 Instituto Dante Pazzanese Instituto de Cardiologia, Nutrologia São PauloSP Brasil Instituto Dante Pazzanese , Instituto de Cardiologia, Nutrologia , São Paulo , SP , Brasil .; 4 Instituto Dante Pazzanese Instituto de Cardiologia Seção de Estatística São PauloSP Brasil Instituto Dante Pazzanese , Instituto de Cardiologia , Seção de Estatística , São Paulo , SP , Brasil .

**Keywords:** Deglutition Disorders, Cardiovascular Diseases, Stroke, Intratracheal Intubation, Malnutrition, Aging

## Abstract

Some conditions consolidated as risk factors for oropharyngeal dysphagia have already been identified in other diseases, such as neurological. Studies on cardiovascular diseases concentrate in individuals in the postoperative period; thus, it is unknown if these same factors occur in individuals hospitalized for clinical or surgical treatment of these diseases.

**Objective:**

to correlate predictive risk factors for oropharyngeal dysphagia in individuals with cardiovascular disease admitted at a reference cardiology hospital.

**Methodology:**

This is a retrospective clinical study. Medical records of 175 individuals hospitalized for clinical and/or surgical treatment at a reference cardiology hospital from January to June 2017, attendants of the Speech-Language Pathology and Nutrition team, were analyzed. Of these, 100 records were included in the study: 41 females and 59 males (mean age 67.56 years). Deaths and individuals from 0 to 18 years were excluded. Stroke, malnutrition, age and prolonged orotracheal intubation were considered predictive risk factors for oropharyngeal dysphagia. Mann-Whitney test and Fisher's test were used for statistical analysis.

**Results:**

Stroke (OR=2.93 p=0.02), malnutrition (OR=2.89 p=0.02) and prolonged orotracheal intubation (OR=3.94 p=0.02) were statistically significant predictors for oropharyngeal dysphagia within this population. Age below 80 years was not significant (p=0.06), but within octogenarians, significance was found (p=0.033).

**Conclusion:**

Stroke, malnutrition, prolonged orotracheal intubation and age > 80 years are predictive risk factors for oropharyngeal dysphagia in adult population with cardiovascular diseases.

## Introduction

Cardiovascular disease is the leading cause of mortality worldwide. ^1 , 2^ Among its treatments, there is a clinical and/or a surgical option, often requiring prolonged hospitalization. Oropharyngeal dysphagia incidence in the population with cardiovascular disease varies from 2.7% to 51%, ^3 , 4^ and most studies have investigated this population within the postoperative period. ^5 , 6^ Regardless of the underlying disease, oropharyngeal dysphagia may be associated with several conditions of hospitalized patients and other clinical conditions associated with cardiovascular disease. ^7 , 8^

General evaluation of hospitalized patients includes nutritional status. Many may present unintentional weight loss due to malnutrition and low muscle strength, increasing the risk of developing oropharyngeal dysphagia. In addition, malnutrition is a frequent complication, as a result of specific difficulties on swallowing, compromising caloric intake levels. ^9^ Some studies proposed the evaluation of nutritional status to predict the risk of dysphagia due to the significant association between these two conditions, in older adults. ^10^ Malnutrition was also recently considered an independent predictor for dysphagia in population with heart failure. ^11 , 12^

Potential risk factors for oropharyngeal dysphagia in cardiovascular disease populations are: advanced age, ^13^ prolonged orotracheal intubation (OTI) ^14 , 15^ and neurological diseases, such as stroke. OTI in cardiac surgery was considered a potential risk to increase the degree of swallowing impairment, ^4^ not only due to the surgical procedure, but also the clinical and respiratory decompensation ^.^ As for stroke, oropharyngeal dysphagia prevalence is high and, although widely studied, ^16 , 17^ it is rarely associated to the population with cardiovascular disease. ^18^

The conditions described above have already been identified as risk factors for oropharyngeal dysphagia. However, in cardiovascular disease, most previous studies include individuals in postoperative period only ^5 , 6 , 14 , 15 , 19^ or report oropharyngeal dysphagia after aortic region impairment. Thus, there are no studies about swallowing disorders during hospitalization for cardiovascular disease treatment, making it necessary to find out whether these predictive risk factors occur in hospitalized patients being medically managed for this disease. Therefore, this study aims to correlate predictive risk factors with oropharyngeal dysphagia in individuals with cardiovascular disease hospitalized at a reference cardiology hospital.

## Methodology

In total, 175 medical charts records of patients with cardiovascular diseases hospitalized for clinical or surgical follow-up at a public reference hospital in cardiology from January to June 2017. All patients were also evaluated by the Speech-Language Pathology and Nutrition team. Of these, 100 records of individuals with oropharyngeal dysphagia were included, 41 females and 59 males (mean age 67.56 years); 75 individuals were excluded due to deaths and age (from 0 to 18 years old). All patients were referred for speech therapy evaluation by the physician who identified some risk for dysphagia, based on risk criteria of the service, and the length of stay ranged from 5 to 40 days. All patients were followed-up by the speech therapy team. Of the patients included, 66 were hospitalized for clinical treatment for several causes, including decompensated heart failure, acute myocardial infarction, stroke, chronic obstructive pulmonary disease (COPD), pneumonia, acute chronic renal disease, infective endocarditis, among others;, and whereas 34 patients were hospitalized for surgical treatment, such as valve, coronary heart disease, vascular surgeries, pacemaker and heart transplantation. Some of the patients submitted to the surgical procedure had a stroke during the intra- and postoperative periods, as described in Table 1 .

**Table 1:** Demographic characteristics of study participants

Clinical Features	Number (N)	Percentage (%)
Hospitalizations for Clinical Treatment	66	66%
Stroke	20	30,30%
Heart failure	16	24,20%
Acute myocardial infarction	7	10,60%
Pneumonia	6	9,10%
Chronic obstructive pulmonary disease	3	4,50%
Others	14	21,20%
Hospitalizations for Surgical Treatment	34	34%
Valve	10	29,40%
Surgery Myocardial Revascularization	9	26,47%
Pacemaker	2	5,80%
Heart Transplantation	2	5,80%
Vascular surgery	5	14,70%
Intraoperative stroke surgery	6	17,60%

This is a retrospective clinical study approved by the Research Ethics Committee of the Institute (Protocol No. 4776/2017). As a retrospective study with database, the informed consent was not required. Data collection on oropharyngeal dysphagia was performed by the analysis of clinical evaluation protocol for oropharyngeal dysphagia of the hospital. It was performed by two Speech-Language pathologists with over 10 years of expertise in dysphagia. Clinical oropharyngeal dysphagia protocol consisted of identification data, personal history, diagnosis, history of pulmonary disease, feeding route, language screening, vocal screening, indirect evaluation by the observation of sensory and orofacial motor performance, and direct evaluation of the biomechanics of swallowing through the use of multiple consistencies and volumes of food. Swallowing was considered functional when the patient did not present impairments in any phases. Oropharyngeal dysphagia was diagnosed when one of the oral and/or pharyngeal phases was impaired, with or without clinical signs suggestive of penetration and/or aspiration (coughing, choking, throat clearing, wet voice), but with nutritional and/or laryngotracheal aspiration risks. Oropharyngeal dysphagia was classified as mild (impairment in oral and/or pharyngeal phases without clinical signs suggestive of penetration and/or aspiration), moderate (impairment in oral and/or pharyngeal phases with clinical signs suggestive of penetration and / or aspiration) or severe (impairment in oral and/or pharyngeal phases with clinical signs suggestive of penetration and/or aspiration in more than one food consistencies). ^16^

Nutritional data were collected by the nutrition team through clinical nutritional assessment protocols, based on body mass index (BMI) classification. ^20^

To analyze predictive risk factors for oropharyngeal dysphagia, parameters were stroke, nutritional status (malnourished, eutrophic and obese), age and OTI time equal to or greater than 48 hours.

## Results

Mann-Whitney’s test and Fisher’s test were used for the statistical analysis of results; and odds ratio statistics (O.R) for categorical data analysis. Univariate and multivariate were used to analyze risk factors associated with dysphagia.

Table 2 shows that 46% of the individuals had oropharyngeal dysphagia and, among these, 58.7% were moderate level.

**Table 2:** Frequency of oropharyngeal dysphagia severity in study population

	Number (N)	Percent (%)
**Oropharyngeal Dysphagia**	46	46.0
Mild	7	15.2
Moderate	27	58.7
Severe	12	26.1

Table 3 shows that 35% of patients were malnourished, 26% had stroke, 39% had prolonged intubation and 82% were over 60 years old.

**Table 3:** Frequency of risk factors for swallowing disorders in study population

Risk Factors		N	%
**Nutrition**	Malnutrition	35	35.0
	Eutrophic	49	49.0
	Overweight / Obesity	16	16.0
**Stroke**	Stroke	26	26.0
**OTI**	Without OTI	43	43.0
	OTI <48 hours	18	18.0
	OTI> 48 hours	39	39.0
**Age**	18 to 59 years	18	18.0
	60 years or more	82	82.0

OTI -Orotracheal Intubation

Risk factors for oropharyngeal dysphagia in cardiovascular diseases

**Figure 1:**
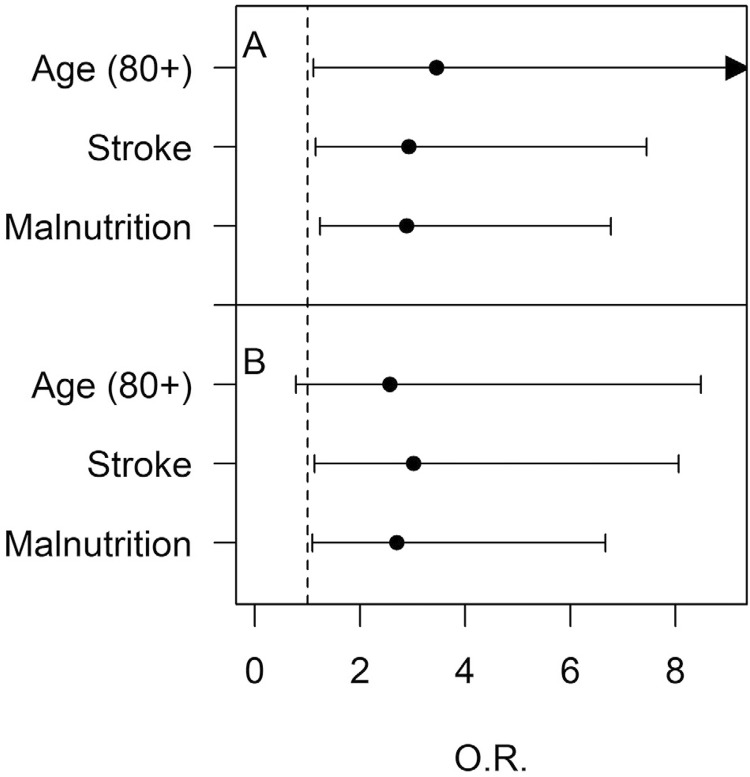
Odds ratios for risk factors associated with oropharyngeal dysphagia in study population OTI -Orotracheal Intubation O.R- Odds Ratio A-Univariate analysis B-Multivariate analysis

Figure 1 shows the association between the presence of oropharyngeal dysphagia and risk factors for swallowing disorders in individuals with cardiovascular diseases. Univariate analysis (A) shows a relationship between dysphagia and malnutrition (OR=2.89 p=0.02) and dysphagia and stroke (OR=2.93 p=0.02). When data were submitted to multivariate analysis (B), a relationship was also identified between dysphagia and malnutrition (OR=2.70 p=0.03) and dysphagia and stroke (OR=3.23 p=0.02). There was no significant relationship between dysphagia and age (p=0.06), but Univariate Analysis showed significance when the octogenarian group was analyzed (OR=3.45 p=0.033) and Multivariate Analysis found values near the significance (OR=2.569, p=0.122). The analysis of the subgroup of patients that have been intubated showedno significant relationship between dysphagia and prolonged orotracheal intubation when considered OTI > 48 hours (OR=2.25 p=0.46), but there was significance when considered OTI> 5 days (OR:3.94 p=0.02).

## Discussion

Investigation about oropharyngeal dysphagia in population with cardiovascular disease is outnumbered when compared to other underlying diseases. Prevalence within literature varies from 2.7% to 51%, which may be related to the method used by each study in the investigation of dysphagia. ^3 , 4^

In this study, 46% of individuals with cardiovascular diseases presented some degree of swallowing impairment. Although this prevalence is within the broad range found in the literature for this population, ^21^ we consider it high. This could be a result of the inclusion of patients not only submitted to surgical procedure, like most of the studies, but in clinical treatment too. Besides that, the high frequency of oropharyngeal dysphagia in our study could be because our hospital possess the previous positive-screening of dysphagia for Speech-Language Pathology assessment. Dysphagia screening can help the team not to miss any patient.

Predictive risk factors for oropharyngeal dysphagia, in this population, that reached statistical significance were stroke, malnutrition and prolonged intubation.

Our findings corroborate with studies which show that stroke is a clinical condition strongly associated with dysphagia. ^17^ The variable incidence in this population is due to the location and extent of the lesion, as well as different investigation methods used, with some studies reporting up to 90% incidence. ^22^ Thus, the presence of oropharyngeal dysphagia in cardiopathy population is closely related to the co-morbidity of stroke, whether or not it is associated with surgery. The generalization of dysphagia in cardiopathy populations deserves further discussion.

Another very important and significant clinical condition in this study was malnutrition. At hospital admission, malnutrition ranges from 20% to 60% and may increase even more in patients with prolonged hospitalizations for clinical and surgical treatment, with a higher incidence among elderly, due to other risk factors associated with this population. ^23 , 24^ According to literature, aging is responsible for the reduction of caloric intake and progressive weight loss, due to catabolic state related to heart failure. Thus, many patients may present nutritional deficits prior to hospitalization and, when combined with undiagnosed dysphagia, it contributes to worsening patient’s prognosis in a hospital setting. ^23^

It is known that oropharyngeal dysphagia can result in malnutrition, what can also be considered a cause of dysphagia. Malnutrition directly impacts the loss of muscle mass, impairing functions of muscles involved in swallowing and breathing and increasing the risk of oropharyngeal dysphagia. ^13^ In our sample, 35% of evaluated patients were malnourished and there was a strong statistical relationship with dysphagia (OR=2.89 p=0.02). Literature reports that 71% of hospitalized patients at risk for dysphagia, regardless of etiology, present alterations in nutritional status. ^25^ Although dysphagia represents an important risk factor for malnutrition, malnutrition effects on swallowing among individuals with cardiovascular diseases remains under study. A recent study with patients with congestive heart failure identified that dementia and malnutrition are independent predictors of dysphagia in elderly people admitted to hospitals: two-third had malnutrition or nutritional risk, and one-third had risk for dysphagia. ^24^

Age was not a significant factor associated with dysphagia in this study, which can be explained by the fact that sample is predominantly made of the older people, as aging is already considered a risk factor for the cardiovascular diseases development ( *p* =0.033). This corroborates with the literature, which states that the progressive physiological aging of structures involved in swallowing process, associated with other comorbidities such as malnutrition, cardiovascular disease, stroke and prolonged hospitalizations negatively impact swallowing, generating risk for oropharyngeal dysphagia. ^13^

Long-term orotracheal intubation, significant in this study when exceeding 5 days and a tracheostomy tube, is considered a predictive risk factor for dysphagia, frequently cited as the cause of it in population submitted to heart surgery. Furthermore, it is extremely important to consider intubation not only within surgical context, but also in patients with cardiopathy hospitalized for clinical treatment, as they may need long periods of intubation. ^4 , 18 , 26 , 27^

The present study has its limitations.It was not possible to categorize possible cardiovascular clinical alterations, as well as the type of cardiac surgery, considering the variability of the sample. In addition, extracorporeal circulation time, the need for transesophageal echocardiography, and associations with other comorbidities such as dyslipidemias, COPD, among others, cited as risk factors for dysphagia in this population, were not analyzed. ^8 , 18^ Another limitation was the method use to evaluate swallowing, since clinical protocols have 73% to 98% accuracy. ^28^ On the other hand, evaluation performed by qualified professionals is able to identify alterations in the physiology of swallowing dynamics, phases involved, degree of impairment, as well as indicate the complementary evaluation, if necessary. Thus, patients with cardiovascular disease admitted for surgical procedures or medical management can present or develop risk factors for dysphagia. This study identified risk factors as: malnutrition, stroke, prolonged intubation and age > 80 years. Patients presenting these should be closely monitored by a multidisciplinary team to minimize possible complications of dysphagia.

## Conclusion

Stroke, malnutrition, prolonged intubation and age > 80 years are predictive risk factors for oropharyngeal dysphagia in adult populations with cardiovascular disease.
